# An efficient functional screening method for anti ATP hydrolase antibody based on fluorescent probes

**DOI:** 10.3389/fimmu.2026.1906242

**Published:** 2026-09-18

**Authors:** Yaofeng Yue, Wei He, Han Yin, Xingnan Li, Huali Shen

**Affiliations:** 1 Institutes of Biomedical Sciences, Fudan University, Shanghai, China; 2 Research and Development Department, Nanjing Zewei Biotechnology Co., Ltd., Nanjing, China

**Keywords:** adenosine triphosphate hydrolase, antibody drug, extracellular nucleoside triphosphate diphosphohydrolase2 (ENTPDase2), functional screening, melanoma mouse tumor model

## Abstract

Antibody drugs as one of the important categories of drugs in the modern biomedicine filed have been successfully applied in various fields, such as cancer diseases and immune disorders. In the early stage of monoclonal antibody drug development, scientists need to accurately select the highly functional antibodies from the numerous candidates obtained from immunized animals for subsequent development. However, due to the diverse binding sites between antigens and antibodies, the high-scoring antibodies obtained through traditional methods, such as enzyme linked immunosorbent assay (ELISA), western blot, surface plasmon resonance (SPR) affinity detection, cannot fully represent their high sealing function towards the target protein. This uncertainty prolongs the research and development cycle and increases inspection costs. This article takes ectonucleoside triphosphate diphosphohydrolase 2 (ENTPD2) as an example to introduce a patented (application number 202511470362.1, China) method for direct antibody screening at the functional level. This screening method mixes the ENTPD2 enzyme, substrate ATP and antibody in a strict dosage ratio to establish precise reaction conditions; upon completion of the reaction, the residual ATP content is detected to evaluate the inhibitory effect of the antibody on the enzyme. To ensure the accuracy of the screening process, we established cell systems and mouse models to verify the function of the antibodies. Using this method, we initially screened 5 antibodies from the supernatants of 214 rabbit monoclonal B cells. These 5 antibodies all demonstrated inhibitory function in both the cell system and the mouse model. These results show that this function screening method possesses the characteristics of high accuracy, high efficiency and high throughput, which can significantly shorten the research and development time and reduce cost.

## Introduction

The ATP molecule is composed of adenine, ribose (5-carbon sugar) and three phosphate groups (**Figure 1**). Among them, adenine and ribose form adenosine, and the three phosphate groups are connected by a phosphate anhydride bond.

**Figure 1:**
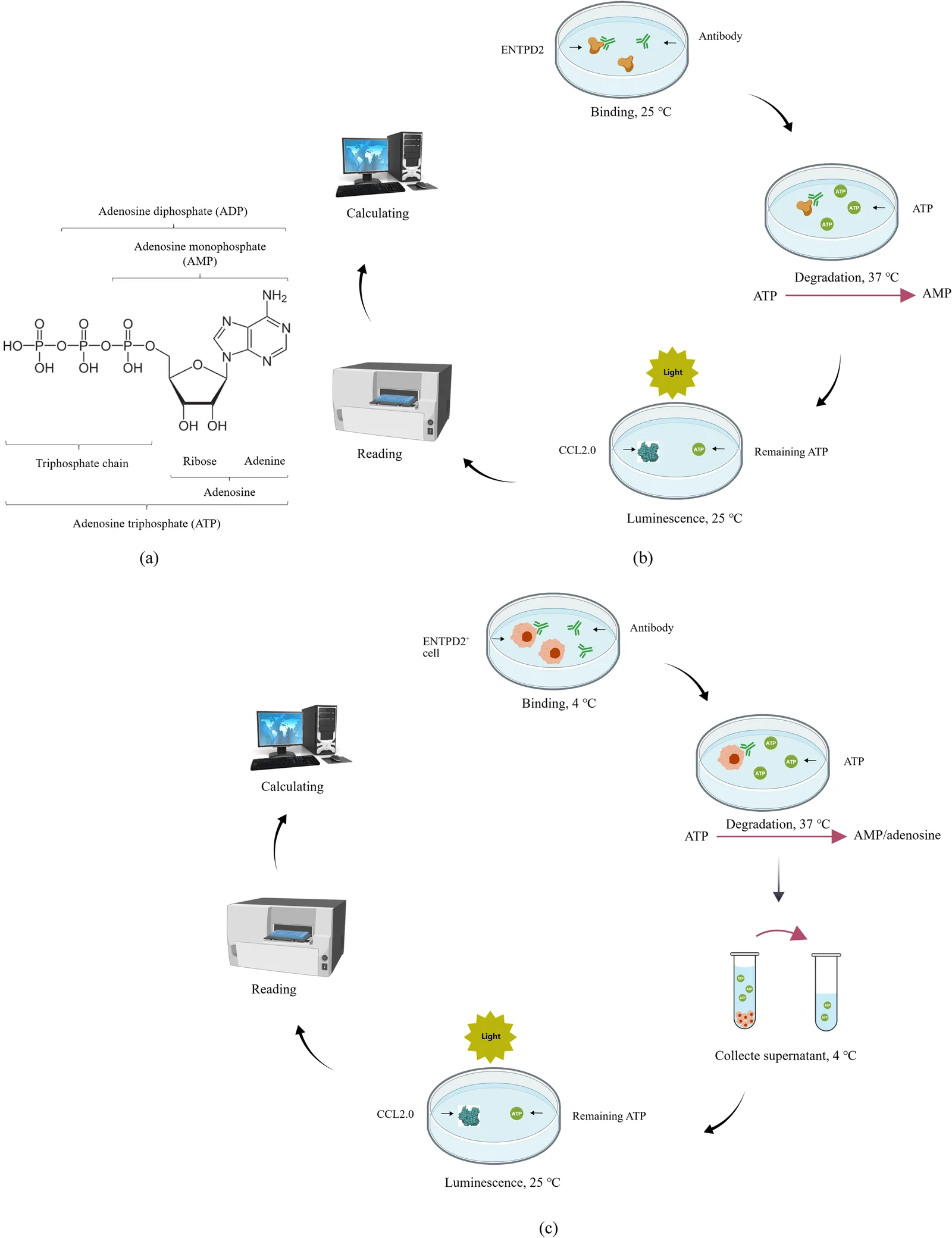
Antibody function screening flowchart. **(A)** The structure of adenosine triphosphate. **(B)** Biochemical system screening process. Created with BioGDP.com. **(C)** Cell verification system process. Created with BioGDP.com.

In living organisms ATP acts as the currency of energy exchange, playing a central connecting role between the processes of energy production and consumption, such as hydrolysis and phosphate group transfer (1). ATP is soluble in water, three phosphate groups can be broken down under the action of enzymes, providing energy for the organism. The energy released when ATP is degraded into ADP can supply all the reactions within the cell. ADP can also be converted back into ATP, providing energy for other intracellular reactions. ATP is also a signaling molecule that is used for cell communication. For instance, a kinase that can phosphorylate molecules can use ATP as the source of the phosphate group (2). Kinases are extremely important for signal transduction, as they can transfer extracellular signals into the cell interior. Once a signal is received within the cell, it will promptly respond appropriately including growth, metabolism, differentiation or apoptosis. The degradation components of ATP are used for gene synthesis. The adenine in ATP is the raw material for the synthesis of ribonucleic acid (RNA) and deoxyribonucleic acid (DNA).

In cells, various enzymes utilize the hydrolysis of ATP to generate energy, which is then used to drive important physiological processes. The enzymes that consume ATP are widely distributed in the cell membrane, cytoplasmic matrix, cytoskeleton, cell nucleus and organelles, and undertake key functions such as ion transport, substance transportation, large molecule synthesis, and energy conversion. Its distribution is closely related to its function, jointly maintaining cellular homeostasis and life activities. According to the distribution location and function of ATP-consuming enzymes, they can be classified into the following categories:

(і) Ion-transporting ATPase. The sodium-potassium ion pump (Na^+^/K^+^-ATPase) maintains the Na^+^/K^+^ concentration gradient on both sides of the cell membrane (3). The calcium ion pump (Ca^+^-ATPase) transports calcium ions (Ca^+^) from the cytoplasm out of the cell or into the endoplasmic reticulum and sarcoplasmic reticulum, participating in the contraction and relaxation functions of muscles. Proton pumps (H^+^-ATPase) transport H^+^ across the membrane to establish a transmembrane proton gradient. (ii) Cellular skeleton related ATPase. Myosin utilizes the energy generated by ATP hydrolysis via its ATPase activity to carry out various life processes, including muscle contraction, cytokinesis, cell migration, intracellular transport, and mechanical signal transduction (4). (iii) Enzymes for the synthesis and modification of biological macromolecules. Aminoacyl-tRNA (transfer RNA) synthetase, DNA ligase and RNA polymerase all require the hydrolysis of ATP to obtain energy (5, 6). (iv) Protein folding and degradation enzymes. Partner proteins assist in protein folding and the ubiquitination regulated proteasome degradation of proteins, all of which require ATP for energy supply (7). (v) Other important ATPase. Helicase unwinds the double stranded DNA to complete DNA replication and transcription. Type II topoisomerases unwind the DNA supercoil. All these activities require the energy provided by ATP hydrolysis (8). (This section of five items was assisted by the AI tool DeepSeek, web version, V3.1.).

ATP provides energy for these normal cellular life activities, enabling them to carry out normal physiological and biochemical processes. The heart, as the continuously working pump organ in the human body, requires a significant amount of ATP for its contraction and relaxation, maintaining the ion gradient across the cell membrane, and other basic metabolic activities. The heart with pathological changes has abnormal ATP consumption. For instance, when a myocardial infraction occurs, ATP synthesis is blocked, resulting in a decrease in myocardial contractility (9).

Furthermore, some studies have reported that there are certain ATP hydrolases on the cell membrane, and their abnormal expression levels are associated with pathological conditions, for instance, the nucleoside triphosphate diphosphohydrolase (NTPDase) family. NTPDase is located on the cell membrane, and its substrates include triphosphate nucleotides and diphosphate nucleotides. In the body, ATP is their primary substrate. The NTPDase family consists of 8 subtypes, all of which are fixed on the cell membrane. They are NTPDase1, 2, 3, 4, 5, 6, 7, and 8. The active domains for ATP hydrolysis of NTPDase4-7 face the inner side of the membrane; while those of NTPDase1-3 and 8 face the outer side (10).

A series of studies have shown that the expression level regulation of ENTPD2 in the NTPDase family is closely related to some disease states. Buffon et al. examined the expression of nucleotide enzymes in Walker 256 mice after tumor cell transplantation on days 6, 10 and 15. They found that each result indicated that *Entpd1 (CD39)*, *Entpd2 (CD39L1)* and *CD73* were the main expressed genes. These genes constituted the main factors of regulatory network and promoted tumor growth (11). Jin Yu et al. reported that when fibrosis and sclerosis occur in the bile duct, bile duct proliferation was upregulated, and during this process, the expression of Entpd2 by portal fibroblasts in the portal area was crucial (12). Srdjan M Vlajkovic et al. discovered that knocking out *Entpd1* in C57BL/6 mice did not affect inner ear function, but the expression levels of *Entpd2* and *Entpd8* in the inner ear increased, suggesting that these two genes metabolically compensated for the effects of *Entpd1* deficiency (13). Aurelie Vandenbeuch et al. reported that after the complete knockout of the Entpd2 gene in mice, the excessive extracellular ATP level in nerve fibers led to insensitivity of P2X receptors, thereby causing a decline in taste responses (14). Belén Ansoleaga et al. reported that, compared with healthy individuals, there was dysregulation of mRNA expression of 10 genes in the olfactory cortex of Alzheimer’s disease patients. Among them, the expression level of *ENTPD2* was significantly increase (15). Benedicto Crespo-Facorro et al. conducted a genetic sequencing analysis on the blood samples of 22 patients with schizophrenia before and after they were administered antipsychotic drugs. They found that before the medication, the expression levels of 6 genes (*ADAMTS2, CD177, CNTNAP3, ENTPD2, RFX2, UNC45B*) significantly increased, and after the medication, these levels decreased to normal levels (16). Kelly Juliana Seibt and colleagues investigated the effects of antipsychotic drugs on the brains of zebrafish. They found that haloperidol reduced the mRNA transcription level of *Entpd2/3*, causing a 35% decrease in ATP hydrolysis, while having no effect on ADP and AMP hydrolysis (17). Andromeda Liñán-Rico et al. isolated and cultured enteric glial cells from 15 gastrointestinal surgery samples. After inducing inflammation with lipopolysaccharide and interferon-γ, they found that the expression of multiple genes, including *ENTPD2*, was upregulated (18). Linda Feldbrügge et al. used dextran sodium sulphate (DSS) to induce colitis in genetically deficient mice. They found that the deficiency of the *Entpd2* gene exacerbated the symptoms of colitis in mice, and the colonic macrophages showed a pro-inflammatory phenotype. The experimental results indicated that Entpd2 has an anti-inflammatory effect on experimental colitis (19). David Kung-Chun Chiu et al. conducted a study using liver cancer models, finding that in the tumor microenvironment, hypoxic conditions induce the increased expression of ENTPD2, thereby enhancing the inhibitory effect of bone marrow-derived suppressor cells on immune cells, ultimately promoting tumor growth; knocking down *ENTPD2* could significantly slow down tumor progression (20). Linda Feldbrügge et al. studied three different mouse liver injury models constructed using various methods and found that mice with the *Entpd2* gene knocked out had a higher degree of fibrosis in liver injury induced by carbon tetrachloride (CCl_4_) (21). Vladimir Grubišić et al. used dextran sodium sulphate to construct an intestinal inflammation model in genetically knockout mice and found that the absence of *Entpd2* increased the permeability of the intestinal tract after inflammation (22). Linda Feldbrügge et al. discovered that complete knockout of *Entpd2* in mice exacerbated the liver toxicity induced by acetaminophen (23). Mafalda S L Aresta Branco et al. investigated the effect of mechanical sensitivity on the function of nucleotide enzymes in mice bladders that degrade ATP and ADP located beneath the basement membrane. The results indicated that these membrane-bound or soluble nucleotide enzymes in the bladder (including ENTPD2) were regulated by mechanical sensitivity to degrade ATP and ADP, thereby reducing the activation of purines at the end of bladder filling. This may be a protective mechanism to prevent excessive bladder activation (24). In the same research focusing on bladder diseases, Alejandro Gutierrez Cruz et al. investigated the regulation of ATP in urine by soluble nucleotide enzymes in the bladder of mice. The results indicated that in case of abnormal bladder excitation, the abnormal release or dysfunction of the soluble nucleotide enzymes (including ENTPD2) in the urinary tract epithelial cells may be the cause of the increased ATP content in the urine (25).

With the emergence and development of bioinformatics analysis methods, a large number of scientific research reports obtained through this method have emerged in recent years. Several bioinformatics analysis studies have indicated that *ENTPD2* is closely related to various diseases and is a key regulatory gene in the development process of these diseases. Chen et al. analyzed the genetic information of 44 liver cancer patients and concluded that the high expression of 5 genes (*ANLN, ENTPD2, TRIP13, PLAC8, G6PD*) in the tumor site was closely related to the poor prognosis of the patients (26). Zhang Jianguo et al. investigated the relationship between the tumor microenvironment of lung adenocarcinoma patients and metabolism-related genes, and finally identified 8 genes (*TYMS, ALDH2, PKM, GNPNAT1, LDHA, ENTPD2, NT5E, MAOB*) that can serve as markers for the diagnosis, monitoring and prognosis assessment of lung adenocarcinoma (27). Cui Lijuan et al. studied the sample information of liver cancer patients and analyzed that five genes, namely *POLA1, UCK2, ACYP1, ENTPD2* and *TXNRD1*, were related to prognosis. The TIMER (tumor immune estimation resource) database indicates that the expression level of ENTPD2 mRNA (messenger RNA) was elevated in liver cancer and was associated with a poor prognosis (28). Chengde Wu et al. investigated the impact of TP53 mutations in the early stage of lung adenocarcinoma on the immune phenotype, and developed an immune prognosis model related to TP53 for evaluating the prognosis of early-stage lung adenocarcinoma. The results showed that two immune-related genes, *ENTPD2* and *MIF*, could best assess the prognosis of patients. In high-risk cases, the expression levels of these two genes were significantly increased, indicating a poorer prognosis and shorter survival period for the patients (29). Wang Zhihao et al. analyzed the prognostic marker genes from 116 metabolic-related genes in lung adenocarcinoma patients, and finally identified 6 genes that could be used to assess the prognosis of lung adenocarcinoma (*ALDOA, catalase (CAT), ENTPD2, GNPNAT1, LDHA, TYMS*) (30). Sanda lacobas et al. conducted a regional gene analysis on the prostate cancer surgical information of a 65-year-old black patient, and identified that four genes, *ENTPD2, AP5M1, BAIAP2L1*, and *TOR1A*, were the key regulatory genes for the main, secondary cancer nodules and the surrounding normal tissues. The expression level of the *ENTPD2* gene was significantly increased in the secondary cancer nodules, while it remained stable in the primary cancer nodules (31). In the same study on prostate cancer, Sanda lacobas et al. developed a bioinformatics algorithm for identifying the unique triad of key regulatory genes in different patients, which was used to develop personalized treatment. Among the sample information of the two tested patients, the analysis result of one of them showed that *ENTPD2, COMMD9* and *MIEN1* were three key control genes with stable expression (32). Lingyan Xiao et al. analyzed the extracellular matrix-related genes of lung adenocarcinoma cells for predicting the survival status of patients, and finally constructed a prognostic feature model based on 8 genes. Among these 8 genes, 6 (*FERMT1*, *CTSV*, *CPS1*, *ENTPD2*, *SERPINB5* and *LYPD3*) were expressed at higher levels in cancer tissues, while the other 2 (*ITGA8* and *ADAMTS8*) were expressed at lower levels (33). Ana Paula Santin Bertoni et al. examined the gene expression levels of the nucleotidease family in rat vascular smooth muscle cells, and conducted a bioinformatics analysis of the expression of these genes in vascular disease models. The results showed that after the vascular lesions occur, *Entpd1*, *Entpd2*, and *Nt5e (CD73)* were more regulated (34). Ali Razaghi et al. conducted a study on the pleural effusion of COVID-19 (coronavirus disease 2019) deceased patients, evaluating 182 protein markers in it. The analysis results showed that the immune systems of the deceased patients were overly activated and triggered severe cytokine storms. This study is the first to report that the expression of *ADA, BTC, DPP6, EDIL3, LIF, ENTPD2, Flt3L* and *LRP1* genes was upregulated in severe patients. In deceased patients, the JAK/STAT pathway was the core regulator of excessive inflammation, and its pathway inhibitors could be potential therapeutic methods (35). Shi Mengchen et al. employed a strategy that combined bioinformatics analysis for screening research targets with experimental investigations to explore the role of ATPase derived from colon cancer cells in the development of colon cancer. It was found that the ENTPD2 enzyme present in exosomes from colon cancer cells promoted ATP -adenosine metabolism, thereby creating an immunosuppressive microenvironment at the tumor site (36).

Further studies have revealed that the expression level of ENTPD2 is in a more central regulatory position within organs such as the heart and blood vessels. B. Rücker et al. conducted a study on the ENTPDase family and CD73 in the left ventricle of rats, and found that the expression level of *Entpd2* was the highest (37). Ana Paula Santin Bertoni et al. discovered that in the synaptic vesicles of rat hearts, the extracellular enzyme with the highest expression level was *Entpd2*, followed by *Entpd6*, indicating its significance in the vascular system (34). Ge Wang et al. employed a method combining bioinformatics with protein mass spectrometry to investigate the mechanism of heart failure development. The results showed that the expression levels of *IMPDH1, ENTPD2, AK7, AK2* and *CANT1* in the left ventricular samples of patients with dilated cardiomyopathy were significantly higher than those in the control group. These genes had the potential to be used as therapeutic targets (38).

These research results indicate that the expression level of ENTPD2 in healthy individuals should be within a normal range. Any deviation from this normal range suggests that the body is experiencing a disease. The deviation in expression levels is either directly caused by the abnormal expression of this gene or is the result of compensatory regulation in response to other gene abnormalities. In either case, ENTPD2 occupies a crucial position in the regulatory network of the disease state, especially in cases of tumor diseases. Whether it is the experimental result reports or the prediction results based on the bioinformatics analysis of clinical samples, they all indicate that the elevated expression of ENTPD2 is closely related to tumor progression. Moreover, there have already experiments showing that reducing the expression of this gene can effectively slow down tumor progression (20). These results suggest that the ENTPD2 target has potential clinical value. Developing drugs targeting this enzyme holds practical significance.

In the field of precision therapy targeting specific molecules, antibody-based drugs are an important category. In the development of antibody drugs, screening functional antibodies is an essential step. The mainstream approach to screening functional antibodies is shifting from the traditional “binding first” model to a more efficient “function first” model (39).

The traditional classical approach centers on the ability to bind. These methods, featuring mature technologies and a long history of application, are currently the most widely adopted antibody screening technologies (40). These methods include: і) hybridoma technology. B cells isolated from the immunized animal are fused with myeloma cells, followed by screening for immortalized cell lines capable of producing the target antibody (41). ii) phage display. The antibody gene is fused with the phage coat protein to achieve its expression on the surface of the phage, and the target antibody is enriched through the “adsorption-elution-amplification” process (42). iii) yeast/mammalian cell display. The principle is analogous to that of phage display, with the key difference being that antibodies are displayed on the surface of yeast or mammalian cells (42). Although these methods are technically mature, their experimental procedures are time-consuming and labor-intensive. Among them, hybridoma technology features a relatively low throughput (43).

The feature of the function-first strategy is that it enables direct screening for activity. This strategy overcomes the drawback inherent in the “binding-first” strategy, where antibodies can achieve binding but do not necessarily exert functional activity. Such methods include: і) microfluidics-based single cell screening. Single cells secreting antibodies are co-encapsulated with reporter cells into microdroplets. If the antibody is functionally active, the reporter cell will generate a detectable signal, such as fluorescence (44, 45). ii) dual-cell screening based on nanovial. Hydrogel nanovials are utilized to encapsulate single antibody-secreting cells and target protein-expressing cells within a confined microscale space. This method enables direct functional screening of antibodies targeting cell membrane proteins (41). iii) cell based functional high throughput screening. For example, by displaying an antibody library on the cell surface and simultaneously detecting downstream signaling pathways, agonist or antagonist antibodies can be directly screened (40).

Furthermore, with the development of artificial intelligence (AI), AI empowerment has been applied in the field of drug discovery and development. і) AI-assisted design and screening. AI can predict key properties of antibodies including structure, stability and affinity, and is also capable of *de novo* designing completely novel antibody sequences. Meanwhile, AI is also capable of analyzing massive datasets and screening out candidate molecules with the highest potential (39, 46). ii) closed loop integration of AI and experiments. AI intelligence is applied for design or preliminary screening, and subsequent validation is performed via high throughput functional experiments. Experimental data are fed back to AI for model training and optimization. Establish an “design-build-test-learn” cycle (39).

In this study, taking ENTPD2 as an example, we reported a method for detecting its ATP hydrolysis capacity at the functional level after being blocked by antibodies. The application scenario mainly lies in the process of developing anti-ENTPD2 antibodies, where the functional activity of the antibodies can be accurately and efficiently measured at the functional level. From the above description, we can understand that ENTPD2 has the potential to serve as a therapeutic target. The drug development strategies targeting this specific target can be (і) small molecule compound inhibitors; (ii) large molecule drugs, which is the antibody described in this study. When the body is stimulated by an immunogen, due to the multi-epitope characteristics of the immunogen, the diversity of B cell receptors, antibody class switching, and polyclonal activation, among other factors, B cells produce a variety of different antibodies, and these antibodies have vastly different functions. Therefore, in the development of antibody drugs, it is necessary to select the effective antibody options from the numerous antibodies initially produced, in order to shorten and reduce the time consumption and research costs of subsequent studies. It can be seen that the accuracy and efficiency of the initial antibody screening in this step are of vital importance to the entire drug development process. Here, we have designed a method to directly verify the blocking effect of the antibody on the ENTPD2 protein at the functional level, in an effort to enhance the accuracy. We used an anti-ENTPD2 antibody that had been reported in patent as the positive control, and verified the antibodies identified by our method in the tumor-bearing mouse model. The data results indicate that this method can accurately and quickly identify highly active antibodies, which is of great benefit to the drug development process.

The methodology reported in this study enables the functional screening of antibody therapeutics targeting the ENTPD2. This method can also be referenced for the screening of small-molecule compound inhibitors targeting this specific target. It only requires replacing the antibody in the system with a small-molecule drug at an appropriate concentration.

## Screening procedure

### Testing the function of anti-human ENTPD2 antibodies in biochemical system

In the initial screening of the biochemical system, in order not to miss any positive antibodies, for the candidate antibody supernatants of unknown concentrations, a repeat screening can be conducted using 5 μL and 15 μL doses. However, the positive antibody mAb1 and the negative antibody mAb13 used a single working concentration and remained constant throughout. The purity of mAb1 and mAb13 antibodies was over 95%.

1) Prepare the reagents.

The B-cell culture medium was heated and inactivated at 60 °C in a water bath for 45 minutes, which was denoted as HI (heat inactivation). If DMEM high glucose medium (Servicebio, G4511) (add 10% foetal bovine serum, FBS, gibco, 16000044) is used, the same method should be adopted for heat inactivation; PBS (phosphate buffer saline) (Servicebio, G4202) does not need to be inactivated. (All the PBS used in this study were free of Ca^2+^ and Mg^2+^).

Dilute the human ENTPD2 protein with PBS to a concentration of 1.667 ng/μL. Place it on ice. Dilute as needed for use.

Dilute the positive control antibody mAb1 (human anti-human ENTPD2, IgG type, patent WO2019/229658A1) in B cell culture medium (HI) (reference materials and methods) to 2 ng/μL (or in HI-DMEM, PBS). Place it on the ice.

Dilute the negative control antibody mAb13 (mouse anti-mouse ENTPD2, IgG type, patent WO2019/229658A1) in B cell culture medium (HI) to 2 ng/μL (or in HI-DMEM, PBS). Place it on the ice.

Dilute ATP (aladdin, A265857) with PBS to a working solution of 333 nM. Place it on the ice.

2) In 96-well microplate, add ENTPD2 protein, PBS, mAb1, mAb13 antibody, the candidate antibody, and solvent (**Table 1**). During the operation, place the microplate on ice to maintain a low temperature.

**Table 1:** Reagent dosages for biochemical system.

	ATP only	ENTPD2+ATP	ENTPD2+ATP+mAb1	ENTPD2+ATP+mAb13	ENTPD2+ATP+candidate antibody
			positive control	negative control	
ENTPD2 protein	—	15 μL	15 μL	15 μL	15 μL
PBS	15 μL	—	—	—	—
mAb1	—	—	15 μL	—	—
mAb13	—	—	—	15 μL	—
Candidate antibody supernatant	—	—	—	—	15 μL (or 5 μL)
Antibody solvent	15 μL	15 μL	—	—	— (or 10 μL)
ATP	40 μL	40 μL	40 μL	40 μL	40 μL
CCL2.0	20 μL	20 μL	20 μL	20 μL	20 μL

At this point, the concentrations of mAb1 and mAb13 antibodies in the system are 1 ng/μL. ENTPD2 protein (mol)/control antibody (mol) ≈ 2.

3) Place the plate in a room temperature environment (25 °C) and incubate for 30 minutes.

4) Place the plate on ice and add 40 μL of ATP working solution to each well.

5) Seal the plate with a sealing film and gently shake it in a 37 °C environment for 40 minutes.

6) Place the plate on ice and add 20 μL of CCL2.0 (reference materials and methods) to each well.

7) Place the plate in a 25 °C environment, shielded from light, and incubate for 10 minutes.

8) Place the plate on ice and check if there are any bubbles in the wells. If so, heat the needle of the syringe over the outer flame of the alcohol lamp and gently pierce the bubble to eliminate it.

NOTE: The bubbles cause the reading value of the instrument to be significantly higher than the actual value.

9) Use an ELISA reader with luciferase fluorescence detection function, such as Victor Nivo, to measure the luminescence value.

10) Calculate the inhibition rate of the antibody on the ENTPD2 enzyme according to the following formula.

The inhibition rate of antibodies on the ENTPD2 enzyme

= (RLUx - RLU1)/(RLU2 - RLU1)

RLU, Relative Light Unit, its value is the luminescence intensity of luciferase fluorescence measured by the ELISA reader.

RLUx: ENTPD2 + ATP + different antibodies

RLU1: ENTPD2 + ATP

RLU2: ATP only

A higher inhibition rate indicates that the antibody has a strong inhibitory effect on the free form of ENTPD2 enzyme.

### Testing the function of anti-human ENTPD2 antibodies in cellular system

In the cell system verification, all the antibodies used were at a single working concentration. The selected antibodies obtained from the initial screening were subjected to large scale expression and purification, and then their functions were verified in cell system. The purity of all antibodies was over 95%.

1) Prepare cells and reagents.

The culture medium DMEM (Servicebio, G4511) (containing 10% FBS, without Penicillin/Streptomycin) was heat-inactivated (HI) at 60 °C for 45 minutes.

Collect the cells (**Tables 2**, **3**) using trypsin digestion method when the confluence reached 70% to 80%. Wash the cell pellet once with PBS. Resuspend the cells in DMEM medium (HI) to a concentration of 1×10^5^ cells/mL. Place it on ice.

**Table 2:** ENTPD2^-^ cell control group and reagent dosage.

	ATP only	ENTPD2^-^ cells+ATP
ENTPD2^-^ cell *	—	100 µL
DMEM HI	165 µL	65 µL
Antibody	—	—
PBS	25 µL	25 µL
ATP	10 µL	10 µL

*ENTPD2^-^ cell: B16F10 mEN KO or HEK293 WT.

**Table 3:** Dosage of ENTPD2^+^ cell control group, test group and reagents.

	OE cells+ATP	OE cells+ATP+mAb1	OE cells+ATP+candidate antibody
		positive control	
OE cell*	100 µL	100 µL	100 µL
DMEM HI	65 µL	65 µL	65 µL
Antibody	—	25 µL	25 µL
PBS	25 µL	—	—
ATP	10 µL	10 µL	10 µL

*OE cell: (cells with overexpression of ENTPD2) B16F10 hEN OE, or HEK293 hEN OE.

The control antibody and the candidate antibody that was highly expressed and purified were dissolved in PBS to a final concentration of 10 ng/μL. Place it on ice.

Dilute ATP with PBS to a concentration of 4440 nM. Place it on ice.

2) Place the 96-well plate on ice and add the cell suspension, DMEM medium (HI), antibodies, and PBS according to the dosages shown in **Tables 2**, **3**. No ATP was added at this time.

At this point, the antibody concentration in the system is 1.3 ng/μL. Cell (mol)/antibody (mol) ≈ 1×10^-18^.

3) Keep the culture plate placed on ice. Gently mix by aspirating and dispensing with a pipette, and then incubate at ice temperature (or 4 °C) for 15 minutes.

4) Place the culture plate on the ice. Add the ATP solution as shown in **Tables 2**, **3**, and gently mix well. Incubate at 37 °C and 5% CO_2_ in incubator for 15 minutes. Remove the culture plate and place it on ice. Use pipette to gently mix it, then return it to incubator for further incubation for 15 minutes.

5) Transfer the culture plate to a pre-cooled 4 °C centrifuge basket and centrifuge for 3 minutes at 300 g.

6) Place the culture plate and ELISA detection plate on ice. Gently transfer 60 μL of the supernatant to the 96-well detection plate. When aspirating the liquid, insert the pipette tip below the liquid surface to approximately 1/2 of the liquid level, and slowly release the pipette button.

Note: Absolute care must be taken to avoid aspirating the cells. Because the incorporated cells would generate a large amount of ATP during the incubation process, the test results would be unreliable.

7) Keep the microplate on ice. Add 20 μL of CCL2.0 (reference materials and methods) to each well (**Table 4**).

**Table 4:** Reaction system in the assay plate.

	Supernatant culture medium	CCL2.0
Volume	60 µL	20 µL

8) Transfer the plate to room temperature environment (25 °C), keep it in the dark and incubate for 10 minutes.

9) Place the plate on ice and check if there are any bubbles in the wells. If so, heat the needle of the syringe over the outer flame of the alcohol lamp and gently pierce the bubble to eliminate it.

10) Use an ELISA reader with luciferase fluorescence detection function to measure the luminescence value.

11) Calculate the inhibition rate of the antibody on the ENTPD2 enzyme according to the following formula.

The inhibition rate of antibodies on the ENTPD2 enzyme on the cell membrane

= (RLUx - RLU1)/(RLU2 - RLU1)

RLU, its value is the luminescence intensity of luciferase fluorescence measured by the ELISA reader.

RLUx: ENTPD2 OE cells + ATP + different antibodies

RLU1: ENTPD2 OE cells + ATP

RLU2: KO cells + ATP

A higher inhibition rate indicates that the antibody has a strong inhibitory effect on the ENTPD2 enzyme on the cell surface.

## Results

### The assessment window of the biochemical system

Luciferase use the energy of ATP to catalyze the substrate luciferin, thereby generating fluorescence. When all other substances are abundant, the content of ATP is positively correlated with the intensity of luminescence, and it shows a linear relationship within a certain range. Based on these principles, we had designed this detection system. As shown in **Figure 2(A)**, the ATP only group did not undergo the reaction of ATP hydrolysis. Its ATP content was the highest, and the measured fluorescence intensity was also the highest. In the ENTPD2 + ATP group, the ATP was hydrolyzed by the ENTPD2 enzyme, resulting in a decrease in its content and a corresponding reduction in the measured fluorescence intensity. The difference in fluorescence intensity between the ATP only group and the ENPTD2 + ATP group represented the detection window of this system. In the positive antibody control group, ENTPD2 + ATP + mAb1, the functional domain of ENTPD2 was blocked by the antibody mAb1, resulting in reduced or abolished ATP degradation capacity. The fluorescence signal intensity generated by the remaining ATP after the reaction ended fell within the detection window. The strong fluorescence signal indicated that the antibody had a strong inhibitory effect on the enzyme. During the early stage of this research, we explored the appropriate dosage concentration. Within this concentration range, the content of ATP was found to have a linear relationship with the luminescence intensity (the data is not presented in this article). Based on these characteristics, we developed a formula for calculating the inhibition rate, which is used for quantitative comparison. From this formula calculation, it can be seen that for the two ends of the detection window, the ATP only group and the ENTPD2 + ATP group, their inhibition rates were 100% and 0% respectively (**Figure 2(B)**). In the positive antibody group, the function of the enzyme was partially inhibited, with an inhibition rate of 84.7%, which fell exactly within the detection window. In the negative antibody control group, the activity of the ENTPD2 enzyme was not affected. The calculated inhibition rate was -0.25%, which could be reasonably regarded as 0%. The results of the antibody control group indicated that this biochemical detection system has reliable specificity.

**Figure 2:**
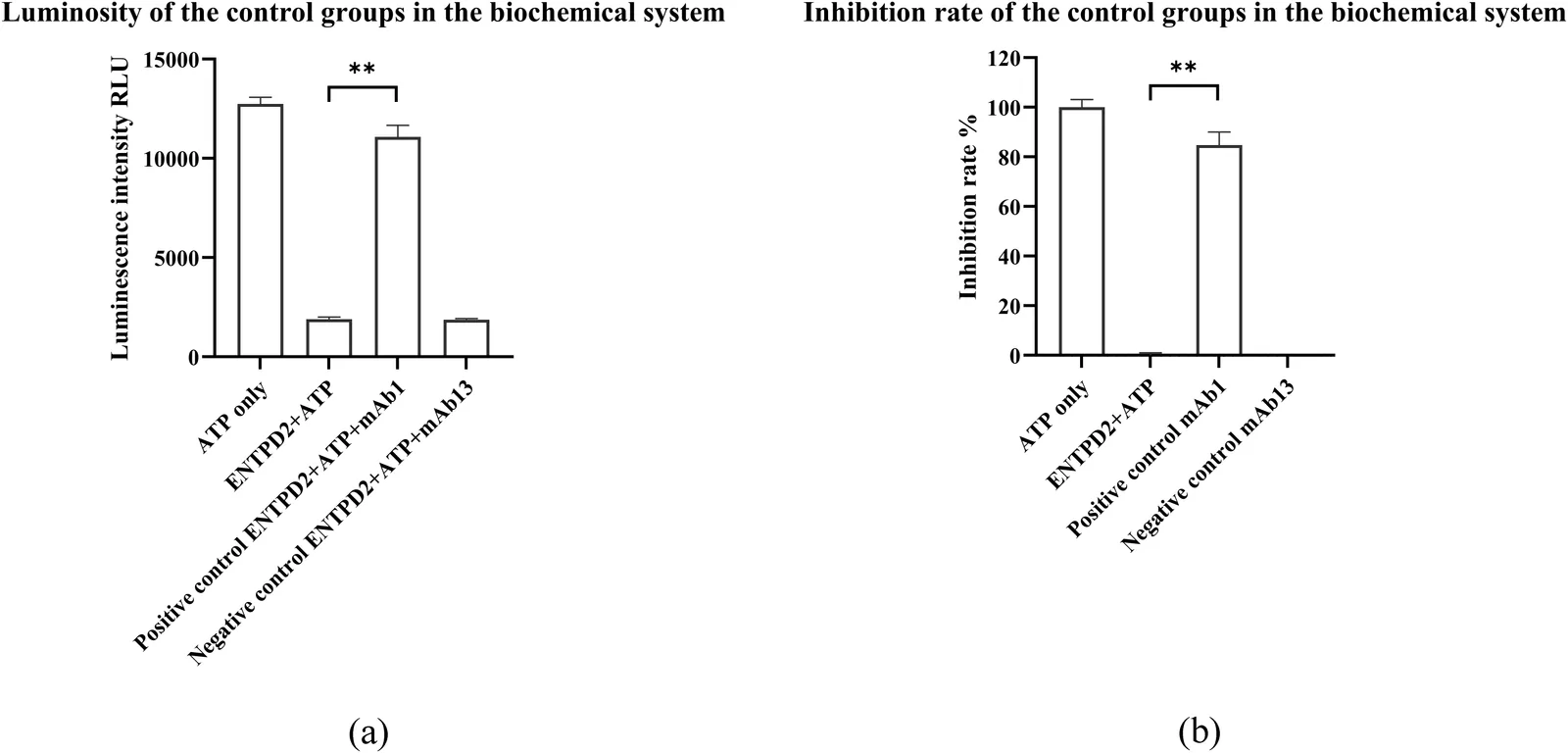
The measurement window of the biochemical detection system and the test results of the control antibody. **(A)** The fluorescence intensity value read by the ELISA reader. **(B)** The inhibition rate is calculated based on the fluorescence intensity values. Error bars: Mean with SEM. Analysis was performed using GraphPad Prism 10 software. Confidence level: 95%. Unpaired t Tests with Welch’s correction, **two-tailed P value < 0.01.

### Antibody screening results of the biochemical system

We utilized a biochemical detection system and selected over 30 antibodies with good inhibitory functions from the supernatants of 214 candidate monoclonal cells using a 15 μL dosage (**Figure 3**).

**Figure 3:**
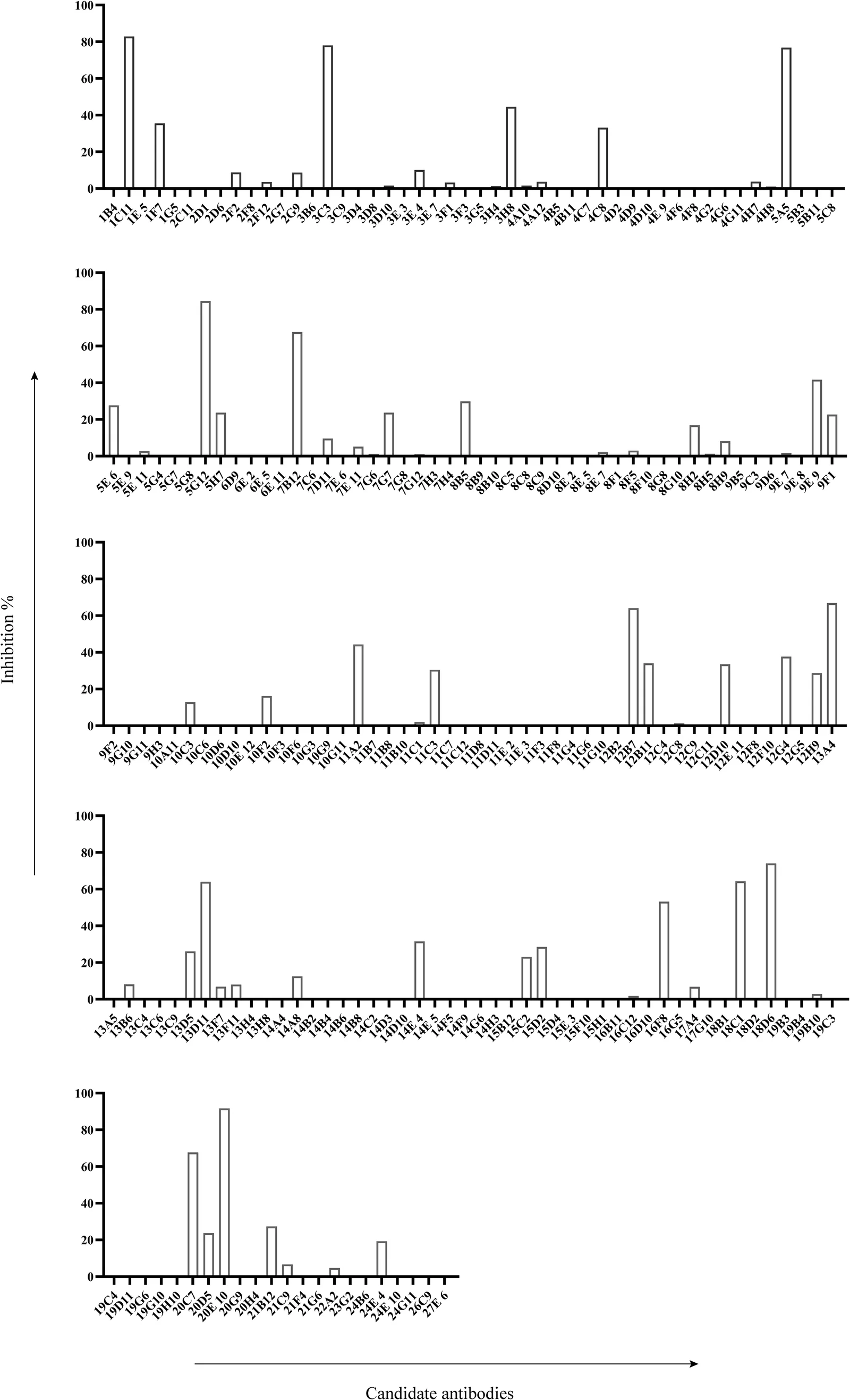
The results of the inhibition rate obtained by screening candidate antibodies using the biochemical system. Based on the biochemical system window shown in **Figure 2A**, the inhibition rates of 214 candidate antibodies against the ENTPD2 enzyme were calculated using the biochemical system formula. Antibody supernatant dosage, 15 μL. X axis, the names of 214 monoclonal antibodies. Y axis, the inhibition rate of antibodies on enzymes.

After several rounds of re-screening, taking into account the functional decline of different antibodies in storage conditions, we selected 5 antibodies, 7B12, 11A2, 12G4, 13A4, 16F8, for large-scale expression and purification, which will be used in subsequent studies.

### The detection window of the cell system and the verification results

Taking the fluorescence intensities measured in the ENTPD2^-^ cell group (KO + ATP) and the ENTPD2^+^ cell group (OE + ATP) as the two endpoints of the detection window, the corresponding inhibition rates were 100% and 0% respectively; the inhibition rate of the antibody group was in between (**Figure 4**). Here, the ENTPD2^-^ cell group was selected as the upper limit endpoint of the detection window instead of the ATP only group. The reasons for this choice are discussed in the following section. From the verification results, the antibodies obtained through the screening of the biochemical detection system, after extensive expression and purification and with a strictly uniform dosage, demonstrated the expected effect in the cell detection system. Here are the test results of the B16F10 mEN KO and B16F10 hEN OE cell systems. The research data indicated that using the HEK293 WT and HEK293 hEN OE cell systems could yield consistent results (this data is not presented in this article). The ELISA detection data of antibodies, please refer to the supplementary material entitled “ELISA assay for antibody binding capacity”.

**Figure 4:**
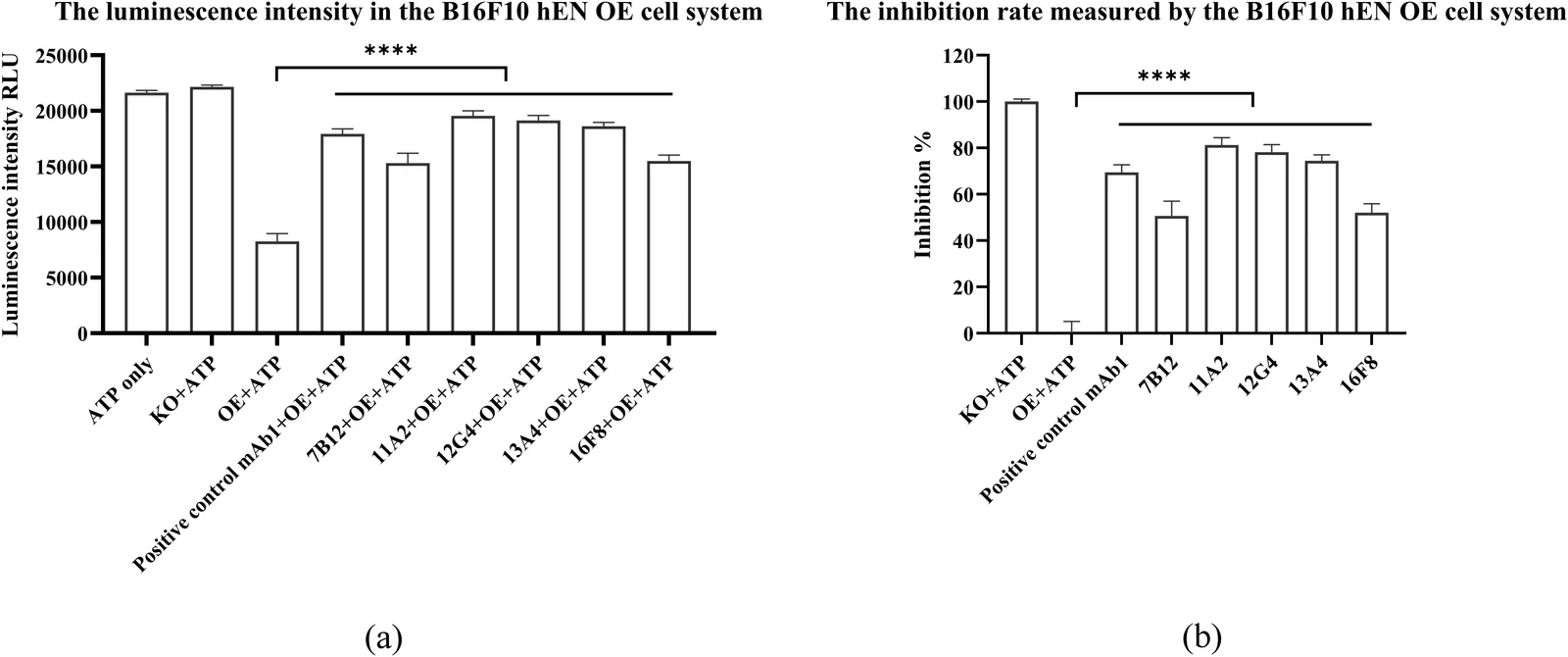
The detection window of the cell system and the antibody verification results. **(A)** The fluorescence intensity value read by the ELISA reader. **(B)** The inhibition rate is calculated based on the fluorescence intensity values. KO: B16F10 mEN KO cell; OE: B16F10 hEN OE cell. Error bars: Mean with SEM. Analysis was performed using GraphPad Prism 10 software. Confidence level: 95%. One-way ANOVA (and nonparametric or mixed), use ordinary ANOVA test, Dunnett Test, 95% confidence interval. ****P vale < 0.0001.

### Verify the function of the antibody in the tumor-bearing mouse model

To verify the function of the antibody more accurately, we subcutaneously inoculated B16F10 hEN OE cells into C57BL/6J mice to establish a tumor model. We use the patented mAb1 antibody as a positive control and conducted *in vivo* experiments according to the treatment protocol in the patent. The results showed that all the antibodies exhibited a trend of inhibiting tumors compared to vehicle; when compared with the positive control mAb1, 7B12 demonstrated a better inhibitory effect (**Figure 5**). This result further validates the effectiveness of these antibodies, and also confirms the validity of this screening method.

**Figure 5:**
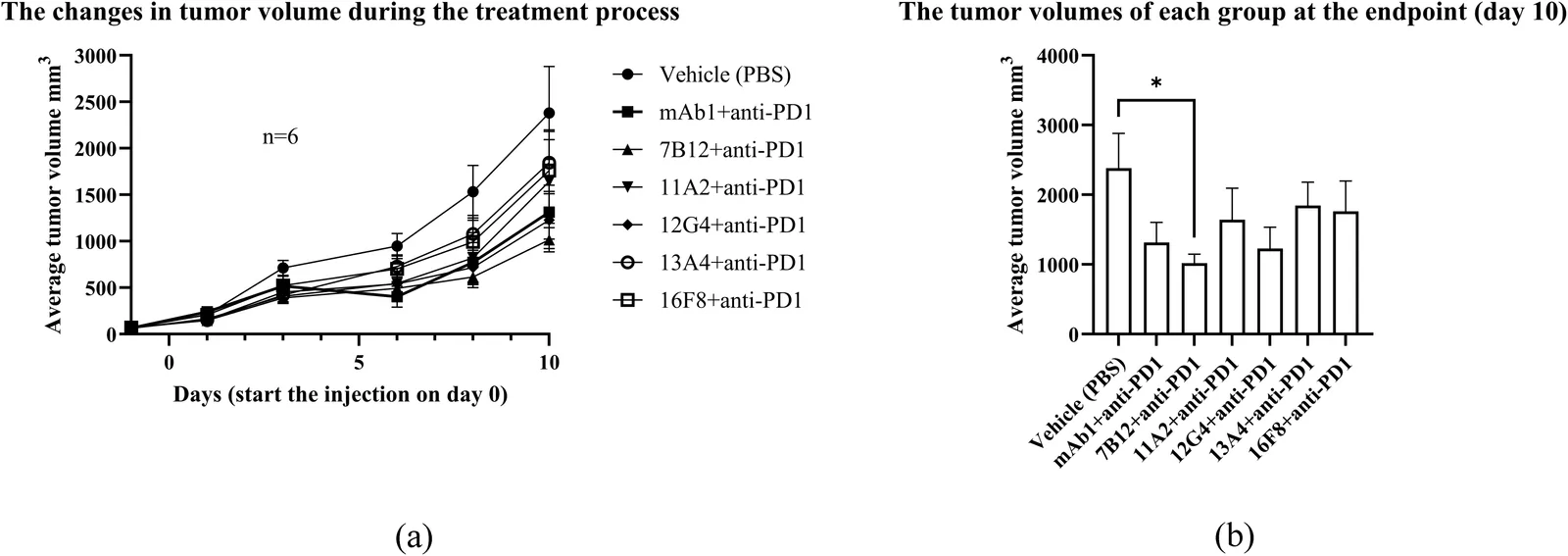
Validate antibody function in a tumor-bearing mouse model (C57BL/6J). **(A)** Tumor growth curves of each group. **(B)** Comparison of tumor volumes at the end of the experiment for each group. Error bars: Mean with SEM. Analysis was performed using GraphPad Prism 10 software. Confidence level: 95%. Unpaired t Tests with Welch’s correction, *two-tailed P value < 0.05. Mouse model sample size was calculated using G*Power 3.1 based on a F test (ANOVA: Repeated measures, within-between interaction), with an effect size f of 0.25, α err prob = 0.05, and power (1-β err prob) = 0.80. This yielded a minimum of 6 mice per group; considering a zero potential attrition rate based on pilot trail. No animals were excluded from the analysis.

## Discussion

In biochemical detection systems, the exact concentration of candidate antibodies was uncertain, and quantitative determination of their concentration presented non-trivial technical challenges. Because the binding abilities of these candidate antibodies to the target antigen (ENTPD2 protein) were not consistent and the difference was significant, they were not suitable for using common detection methods that rely on the binding of antigen and antibody, such as ELISA, western blotting, and SPR affinity determination. These traditional detection methods can, to a certain extent, distinguish the strength of binding ability. However, they were still far from achieving the goal of being economical and efficient. Meanwhile, the candidate antibodies were dissolved in the cell culture medium, and the complex substances contained therein have an impact on the precise quantification that cannot be ignored. Therefore, we will directly consider it from the functional perspective. The function of the ENTPD2 in the body is to degrade ATP, and this process takes in a complex environment. Functional indicators are the final useful information in a complex environment. The biochemical detection system we have developed eliminated the need for purifying antibodies, which significantly reduced the cost.

On the other hand, our previous data also demonstrated that antibodies with higher affinity did not necessarily exhibit stronger inhibitory effects on hydrolytic activity. The ranking of binding affinity was not consistent with that of inhibitory function (this data is not shown in this article). The reasonable explanation for this is that the positions where the antigens bind to different antibodies are different. Some antibodies, although having high affinity and strong binding ability to the antigen, fail to effectively block the functional domain that hydrolyzes ATP due to their positioning. As a result, they exhibit relatively low inhibitory functions. While traditional detection methods only assess the binding force and cannot distinguish the blocking ability for functional domains. The antibodies selected in these ways have poor accuracy. Subsequently, a large amount of expression, purification of the antibodies, and re-screening in animal models are required, which increases the research and development costs.

Consistency in candidate antibody concentration was achieved through consistent cultivation processes, such as uniform culture conditions and incubation times, as well as consistent sample volumes during final testing. Although this did not completely ensure consistent secretion concentration for each antibody, it did not affect qualitative assessment. For some candidate antibodies, it might be because the sampling concentration was too low that they were not selected. For such situations, we adopted the strategy of increasing the concentration and conducting repeated tests. First, used 5 μL for the test, then 15 μL. If after increasing the dosage, the function of degrading ATP still did not appear, then it could be concluded that the antibody secreted by this monoclonal cell had no function or a very low function. If it did not meet the requirements, it could be eliminated. In fact, based on the test results, a 5 μL volume of antibody with good functionality was sufficient to demonstrate inhibition of ENTPD2 enzyme activity. To maximize the accuracy of initial screening, repeating the process using this method still offered significant economic advantage. Additionally, to provide optimal reaction conditions as much as possible, we dissolved and diluted the antibodies in heat-inactivated B cell culture medium, thereby introducing a small amount of medium into the system to simulate the native secretion environment of the antibody. The data results indicated that this had some benefits in expanding the detection window, but it was not a necessary factor. The operator can choose one of the three solvents (B-cell culture medium, PBS, or DMEM medium) according to the conditions of their laboratory.

In the biochemical detection system, the culture medium contains FBS, and the complement components present in it will bind to the Fc segment of IgG antibodies, thereby interfering with the detection. Similarly, such binding also exists in cell detection system and may cause significant interference. Because the complement-dependent cytotoxicity can lead to cell lysis, thereby disrupting the detection system. Therefore, we pre-treated the culture medium with heat to inactive the complement, thereby eliminating potential adverse factors.

Regarding the detection in the cellular system, we utilized transgenic cells HEK293 hEN OE derived from human embryonic kidney and transgenic cells B16F10 hEN OE derived from mouse melanoma. From the results, the detection outcomes of these two cell systems were consistent, and both could be used to detect the function of ENTPD2 in degrading ATP. The former features lower construction costs, as it eliminates the intermediate step of gene knockout. Its control cells could simply use wild-type HEK293. The construction of the latter involves the knockout of the mouse-derived *Entpd2*, which complicates the workflow. Users can make the best choice based on their actual usage scenarios.

The biochemical detection system here can be said to have more precisely established a system for testing the blocking effect of antibodies on the degradation of ATP by the enzyme. Since there are no complex components such as cells or tissues in the system, the focus is solely on the relationship among the three elements: antibodies, enzymes, and substrate ATP. In addition to having a short detection time and being convenient and fast, this system also has a very practical advantage – it requires a small amount of antibody. Only 5-15 μL of the original B cell culture supernatant is needed, without the need for large-scale antibody expression, purification, as well as traditional testing. Therefore, it is highly suitable for the initial screening of antibodies, and it can achieve high-throughput screening. It can quickly select the functional options from a large number of monoclonal cells. Because the blocking function of the antibodies is directly used as the evaluation criterion, the screening results are also highly accurate. In the cell testing system, the participation of cells, which are the basic units of life structure and function, makes it closer to the real environment. For instance, the ENTPD2 enzyme is a transmembrane protein with its catalytic region facing outward, which is different from proteins that are completely in a free state in the biochemical system. Therefore, this cell system can be utilized to further validate the antibodies selected through the biochemical screening process. However, one characteristic of the cell system is that it requires a large amount of antibody, which limits its application as a large-scale initial screening method. We used it as a verification of the initial screening results, and the outcome proved that this was a satisfactory choice. After all, the selected antibodies will eventually be used for *in vivo* treatment. Guided by this goal, we need to know whether these antibodies still retain their functions in the body. Currently, the most suitable method for simulating the *in vivo* environment is still animal experiments. However, it is obvious that this requires a significant investment of resources and time, as well as a much larger dose of antibodies. Compared to animal testing, using cell-based system for functional validation of large-scale expressed and purified antibodies is also a suitable option. Compared to animal experiments, cell-based systems significantly less antibody and shorter detection times. Compared to biochemical system, the inclusion of cells brings the system closer to the real *in vivo* environment. If the sole purpose is to assess the inhibitory function of antibodies on enzymes, both biochemical systems and cellular systems can handle the task. In living organisms, multiple systems work together to form a complex regulatory network. As mentioned earlier, the effect of ENTPD2 on tumor development in the body is achieved through the regulation of the immune system (20). In this study, in order to assess the function of the antibodies more accurately, we also conducted animal experiments and established a mouse tumor model to verify the function of the antibodies. The results showed that monoclonal antibodies initially screened in biochemical systems also exhibited inhibitory effects in cellular system and demonstrated a trend toward tumor suppression *in vivo*. These results indicate that the antibody screening method reported in this study is an economical, efficient and accurate approach, which can significantly reduce the initial research and development costs of antibody drugs.

In terms of experimental manipulation, the cell is an extremely complex unit of life. We believe that even in modern times, with greatly advanced technology, humans have not yet fully understood cells. One piece of evidence is that humans have only limited types of diseases they can completely cure. Many diseases cannot be cured, such as advanced cancer, acquired immunodeficiency syndrome (AIDS), and even hepatitis B. So far, the medical community still lacks a way to completely eliminate the hepatitis B virus (HBV) from chronic patients’ bodies and can only strive for functional cure. These points are mentioned to clarify that a cell is an extremely complex structure in which numerous known and unknown biochemical reactions take place. We decided to investigate specifically the ATP hydrolysis step during the detection process because changes in the catalytic activity of ENTPD2 constitute the core of this research, and other metabolic activities would interfere with the investigation of this target. Temperature control can eliminate such interferences. Temperature is an important environmental factor, and low temperatures can inhibit intracellular biochemical reactions. Since ENTPD2 is overexpressed, after low-temperature incubation, it quickly exhibits a cumulative hydrolytic effect during the brief 37 °C reaction period, while other biochemical reactions have not yet fully recovered. This strategy effectively minimizes interference from other reactions, highlighting only the interaction between ENTPD2, antibody, and ATP. For this characteristic, we identified an appropriate temperature control method and reaction time in our study, which effectively reduces the background. In biochemical systems, precise control of temperature and time is also required. In biochemical systems, the hydrolysis of ATP is a rapid process, whereas sample addition is time-consuming, especially when dealing with multiple samples. At a room temperature of 25 °C, it is evident that there is a significant difference in reaction time between the first and the last sample; comparing them together has no meaningful reference value. Low temperatures can suspend the catalytic function of enzymes, and more importantly, this inhibition is reversible. Therefore, we adopted a method of lowering the temperature and precisely controlling the time to maintain consistent reaction duration and intensity across all samples, while also ensuring the inhibition rate falls within an appropriate detection range. This is meaningful for comparing the functional differences among various antibodies. In fact, there is also a need to standardize reaction time and intensity in cellular systems, which can be effectively addressed through precise control of temperature and time. Accordingly, in practical applications, operators shall conduct testing in accordance with process specifications and exercise strict control over these two parameters.

What is the appropriate temperature? J.S. Willis et al. reported that the activity of the Na-K pump in human red blood cells at 5 °C was only 0.25% of that at 37 °C (47, 48). In our actual operation, we also found that storing the biochemical or cellular systems at 0 °C (on the surface of crushed ice) or 4 °C (in a refrigerator) for several hours has no significant effect on the systems. This period of time is sufficient for completing the sample addition process. However, if the cell system was exposed to a temperature of 25 °C (room temperature environment), it only took a few minutes for the cells to produce a large amount of ATP. The production volume was much higher than the consumption of the surface protein ENTPD2, which resulted in a significant increase in background signals and affected the analysis. At 25 °C, the ENTPD2 in biochemical system canned also rapidly degrade a large amount of ATP, resulting in inconsistent initial ATP levels in different samples.

Regarding the establishment of the inhibition rate calculation formula that was involved in both processes, we conducted a series of dose explorations in the early stage (this data is not presented in the article). The concentration of ATP is positively correlated with the intensity of the emitted fluorescence. However, only when the ATP concentration is within a certain range will there be a linear relationship between it and the fluorescence intensity. And the calculation formula we established is meaningful only in the context of linear relationships. On the other hand, it concerns the setting of the detection window. As can be seen from **Figure 2**, the difference in fluorescence intensity between the ATP only group and the ENTPD2 + ATP group of the biochemical system constitutes the detection window. This window needs to be as wide as possible and within an appropriate signal strength range in order to better distinguish different antibodies. This requires regulation through the dosage of the ENTPD2 enzyme and the concentration of ATP. If this window is not set properly, two extreme situations will occur. The first phenomenon is that the calculated inhibition rate of most antibodies is 0%. There are two reasons for this outcome: і) These antibodies have no inhibitory function, and therefore their inhibition rate is naturally 0%. ii) These antibodies have slightly lower inhibitory functions. However, in this system, there is an excessive abundance of ENTPD2 enzyme or an insufficient concentration of the substrate ATP. In either case, ATP will be depleted prematurely, leading to the false result of a 0% inhibition rate in calculation, which is a false negative. Of course, in practice, as long as there are still some antibodies for which the calculated inhibition rate is greater than 0%, these apparent 0% inhibition rates will not affect our selection, unless we wish to increase the number of positive results. Since we only want antibodies with strong inhibitory function, those with poor function can be disregarded. The second phenomenon is that some antibodies yield an inhibition rate of 100% in calculations. These antibodies are indeed those with relatively high inhibitory functions. However, from the perspective of system dosage, this is because there is insufficient ENTPD2 enzyme or excessive substrate ATP, resulting in a large amount of ATP remaining after the reaction. In this case, we cannot distinguish which of these 100% inhibitory antibodies has a better inhibitory function. We can consider all these antibodies as qualified candidates. However, when we aim to further reduce the number of positives in order to minimize future investments, this data can no longer provide additional assistance. Therefore, when setting the detection window, our criterion for selecting the dose is: the antibody with the best effect in the calculated inhibition rate showed an inhibition rate within the range of 50% to 100%, while also maintaining ATP concentration within the linear range. In the cellular system, the detection window is the difference in signal intensity between the ENTPD2^-^ cells + ATP group and the ENTPD2^+^ cells + ATP group. In this system, it is also necessary to find the appropriate ATP concentration and cell count. One point that still needs to be emphasized in the cell detection system is the control of interfering factors. Our preliminary experimental data show that cells produced large amounts of ATP after they begin to adhere, resulting in a strong background signal that affected assessment. To address this issue, we adopted the following strategy: і) Use non-tissue culture treated reaction plate to reduce adhesion induction; ii) Strictly control the reaction temperature and time. For the upper limit of the detection window in the cell system, we chose the ENTPD2^-^ cells + ATP group rather than the ATP only group, primarily because other reactions exist within the cells. These reactions, although unrelated to the detection target, can cause significant changes in ATP levels within the system.

We also observed that the ranking of inhibition rates obtained for the six selected antibodies in cell-based and *in vivo* assays was not entirely consistent. The main reasons are primarily considered to be these factors: the complex *in vivo* environment and the stability of the antibodies themselves. From mechanistic studies on antibody-mediated tumor inhibition, we have learned that the immune system plays a crucial role in this process. C57BL/6J is an immunocompetent mouse strain. Existing studies have reported that the anti-tumor capacity of the immune system varies substantially among different individuals of the same species (49). Moreover, different antibodies may vary in stability during storage and in the *in vivo* environment, and these combined factors can lead to discrepancies between the rankings of results obtained from cell-based systems and those from *in vivo* assays. However, from a qualitative standpoint, it can be determined that these antibodies all have a tendency to suppress tumors *in vivo*.

## Materials and methods

### Antibody solvent

B cell culture medium:

RPMI 1640 (Procell system, PM150140) (containing 2 mM L-glutamine, 10 mM HEPES, 1 mM sodium pyruvate) was supplemented with 5% FBS (gibco, 16000044), 55 µM β-mercaptoethanol (aladdin, M301574), 1% MEM non-essential amino acids (Procell system, PB180424), IL-2 (50 ng/mL) (R&D Systems, 6994-R2), IL-4 (10 ng/mL) (R&D Systems, 6939-RB), IL-21 (10 ng/mL) (R&D Systems, 7274-RB) and B-cell activating factor (BAFF) (10 ng/mL) (AmyJet, BGT-PCT-156).

All the concentrations mentioned above were the final concentrations.

Note: The B-cell culture medium can also be replaced by PBS or DMEM medium (Servicebio, G4511) (containing 10% FBS and without Penicillin/Streptomycin) to reduce costs. The experimental results showed that the detection window in the systems using PBS or DMEM as substitutes was slightly narrower, but this did not affect the evaluation of the results.

### Antibody and enzyme

Candidate antibodies: After immunizing rabbit with human ENTPD2 protein (R&D Systems, 6087-EN), 214 B-cell monoclonal cell culture supernatants (containing IgG-type antibodies) were obtained. The same batch of tests used the same dosage, ranging from 5 μL to 15 μL. To reduce the dosage, make up the volume with PBS. As shown in **Table 1**, 15 μL of the supernatant from each antibody was used. If it was reduced to 5 μL (including the control antibody), then 10 μL of PBS was added to the system.

ATP hydrolase: the biochemical system used the same ENTPD2 protein (R&D Systems, 6087-EN).

### Reagents and detection plates

CellCounting-Lite^®^ 2.0 Luminescent Cell Viability Assay (CCL2.0) (Vazyme, DD1101). CCL2.0 is a cell viability detection reagent based on the luciferase system. It contains high purity luciferin and thermostable luciferase. Luciferase consumes luciferin and ATP to emit stable, glow type fluorescence. The light intensity has a linear relationship with the ATP concentration within a certain range. Therefore, this reagent can be used for quantitative detection of the number of living cells. In this study, we directly quantitatively determined the ATP concentration in the solution using the CCL2.0 reagent. Determine the linear range by adjusting the concentration of reagents in the system, and analyze ATP content in different samples.

96 well plate, without tissue culture treatment (NEST, 701012).

96-well ELISA detection plate, black flat bottom (corning, 3916).

### Cell

Cells with overexpressed ENTPD2 (I). Cells constructed through genetic modification based on mouse melanoma cells B16F10. a) Knock out the *Entpd2* gene in the wild-type B16F10 cell, and it was recorded as B16F10 mEN KO. b) The human *ENTPD2* gene was overexpressed in B16F10 mEN KO cell, and it was recorded as B16F10 hEN OE. When using B16F10 hEN OE as the experimental group, the negative control cell was B16F10 mEN KO.

Wild type (WT) B16F10 cells express murine ENTPD2 protein, which also degrades ATP and will interfere with the detection system. We knocked out the mouse ENTPD2 and overexpressed the human ENTPD2. Human ENTPD2 can bind to anti-human ENTPD2 protein, which is consistent with the detection purpose. We used CRISPR/Cas9 to knockout the mouse ENTPD2 and employed a lentiviral delivery system to overexpress the human ENTPD2. Refer to the supplementary file “Construct B16F10 hEN OE cell”.

Cells with overexpressed ENTPD2 (II). The human *ENTPD2* gene was overexpressed in human embryonic kidney cells HEK293, which was designated as HEK293 hEN OE. When using HEK293 hEN OE as the experimental group, the negative control cells were wild-type HEK293. The overexpression tool was the same as that used for B16F10 hEN OE.

### Establish mouse tumor model

We established syngeneic transplantation model using subcutaneous inoculation. The function of anti-ENTPD2 antibodies in inhibiting tumors requires the involvement of the immune system. The B16F10 melanoma cells originate from C57BL/6 mice, which are immunocompetent mice. Transplanting B16F10 cells into C57BL/6 mice avoids immune rejection.

Mouse: C57BL/6J, ♀, at the age of 7 to 8 weeks when inoculating cells.

Cell: B16F10 hEN OE.

Cell culture medium: DMEM (Servicebio, G4511) (glucose 4500 mg/L) + 10% FBS (gibco, 16000-044) + 1% Penicillin/Streptomycin (Servicebio, G4003). Add puromycin (YEASEN, 60210ES25) with final concentration of 5 μg/mL (for screening purposes).

Procedure:

1) Day0, preoperative skin preparation. After the quarantine period, transfer the mice to the workbench. For right-handed individuals, grasp the mouse by gently pinching the skin at the back of its neck with the thumb and index finger of the left hand. Press the hind limbs and tail of the mouse onto the palm using the left ring finger to secure it in place. Expose the right ribcage of the mouse. Hold the trimmer in your right hand and shave from the right side of the abdomen up to the axilla. Approximately 3 cm × 1 cm in length and width, with the long axis extending from the right abdomen to the axilla. Apply a small amount of hair removal cream (brand, Veet) to this haircut area using a cotton swab, then rinse thoroughly with warm water after about 2 minutes. Use sterile paper to absorb the moisture from the skin surface. Place the mouse back into the cage for continued housing.

2) Day 1, prepare cells. For example, using a T25 culture flask, collect the cells when they reach 80% confluence. After removing the medium, wash the cells with 5 mL PBS and then remove the PBS. Add 1 mL of 0.25% trypsin-EDTA (ThermerFisher, 25200056), enough to cover the cells. Digest for approximately 2 minutes at room temperature, and stop the digestion when cells shrink and round up under the microscope. Remove trypsin, add 5 mL of medium containing 10% FBS, and gently tap the edges of the culture flask to detach the cells. Transfer the cell suspension to a centrifuge tube and centrifuge at 4 °C, 300g for 3 minutes to remove the culture medium. Resuspend the cells in 5 mL of cold PBS. Take 18 μL of cell suspension and add 2 μL of Trypan Blue reagent, then mix thoroughly. Transfer 10 μL of the stained sample to the counting chamber of a hemocytometer and count the colorless live cells. If the total number of cells in the large square of the counting chamber exceeds 300, dilute the sample with PBS and recount. Centrifuge to remove PBS, then resuspend the cells in cold PBS according to the count to a concentration of 1×10^7^ cells/mL. Place the cells on crushed ice and immediately transfer them to the mouse workbench.

Note: It is very important to maintain high cell viability. Improper procedures such as excessive digestion or mechanical damage can affect cell viability, thereby influencing tumor growth.

3) Subcutaneous injection. Gently swirl to mix the cells. Use a 1 mL syringe to draw up 50 μL of suspension (containing 5×10^5^ cells). Wipe the needle with an alcohol swab to remove residual cells, preventing them from remaining in the insertion channel and causing irregular tumor shape. Hold the mouse with the left hand as during skin preparation, and use an alcohol swab in the right hand to disinfect the injection site. Wait approximately 10 seconds for the alcohol to evaporate before beginning the injection. Hold the syringe in your right hand with the needle bevel facing upward, and insert it into skin at the lower part of the hairless area. Insert the needle at an angle of approximately 30° into the skin, entering the subcutaneous space between the skin and muscle, without piercing into the underlying muscle. The force feedback from the syringe can be used to determine whether the needle has penetrated the skin; once penetration is detected stop puncturing immediately. Then adjust the syringe so that it is parallel to the skin surface, and use your right thumb and index finger to grasp the outer side of the syringe, advancing it forward. Gently lift the needle tip upward while advancing to avoid puncturing the muscle. Advance the needle approximately 1.0-1.5 cm, then stop. Hold the syringe steady with the right index and middle fingers, and gently push the plunger with the thumb to inject the cell suspension into the subcutaneous space. A circular bulge will form here. Retract the needle. Use your right thumb and index finger to grasp the outside of the syringe and pull it out. While withdrawing the needle, rotate the syringe counterclockwise to prevent the cell suspension from leaking along the needle track. Press the needle site with a dry cotton swab to prevent leakage. Once the needle puncture site has contracted and stabilized, return the mouse to its cage.

4) Observation and measurement. Observe the mice daily. The cells will form a lump in about four days. Measure tumor volume using caliper.

Formula for calculating the tumor volume of mice:

V = (L×W^2^)/2

V: tumor volume (mm^3^)

L: major axis, the longest diameter (mm)

W: minor axis, the diameter perpendicular to L (mm)

By day 7, the tumor volume will reach 30-50 mm^3^. Start injecting the antibodies.

### Mouse *in vivo* treatment trial

Melt the frozen antibodies on ice. Dilute the antibody to 2 μg/μL using cold PBS, place it on ice and transfer it to the animal room. Human-derived anti-human ENTPD2 antibody (positive control mAb1) and rabbit-derived anti-human ENTPD2 antibody (candidate antibody), at a dose of 10 mg/kg, administered by tail vein injection. Mouse-derived anti-mouse PD1 antibody (abinvivo, B176401), at a dose of 10 mg/kg, administered by intraperitoneal injection. In the control group, PBS was used instead of the antibody, with the same volume.

Inject the antibody at the time indicated in **Table 5**. Inject anti-ENTPD2 antibody once. Two injections of anti-PD1 antibody were administered with a 3-day interval in between. Before starting the injection of the antibodies, the initial body weight and tumor volume of the mice were measured. Monitor body weight and tumor volume every day. Approximately 9-10 days after the start of antibody injection, the experimental endpoint was reached and all the mice were euthanized.

**Table 5:** Grouping of mouse experiments and antibody injection times.

Groups	Anti-ENTPD2 antibody (i.v.)*	Anti-PD1 antibody (i.p.)**
Vehicle (PBS)		
mAb1 + anti-PD1 (positive control)	day1	day1, day5
Candidate anti-ENPTD2 + anti-PD1	day1	day1, day5

*(i.v.): intravenous injection; **(i.p.): intraperitoneal injection.

All animal procedures were conducted in accordance with the National Institutes of Health Guidelines for the Care and Use of Laboratory Animals. All animal experiments were approved by the Immunophage Biotech Institutional Animal Care and Use Committee (IACUC, ZW-T2).

## Data Availability

The original contributions presented in the study are included in the article/supplementary material. Further inquiries can be directed to the corresponding author.
